# Quality improvement project: delirium awareness and training in coventry memory services

**DOI:** 10.1192/bjo.2021.610

**Published:** 2021-06-18

**Authors:** Nurul Yahya, Karim Saad

**Affiliations:** Coventry and Warwickshire Partnership Trust

## Abstract

**Aims:**

By way of Quality Improvement, this project aims to identify awareness levels, deliver a brief training and thus increasing the confidence of Memory Assessment Clinicians in detecting delirium.

**Background:**

People with dementia are at greater risk of delirium, and the acute confusion associated with delirium may be mistaken as part of their dementia. Despite having an estimated prevalence in care homes of 14.2% in the UK, delirium is under-recognised. Memory Assessment Clinicians may have low confidence in identifying and have low awareness of delirium despite being tasked with a triage and diagnostic role in dementia assessment. NICE has recently updated the guidelines on Delirium in March 2019 with recommendations on prevention and treatment of Delirium.

**Method:**

We delivered a survey pertaining:

- Awareness of Delirium NICE Guidelines
- Confidence in spotting Delirium

We used convenience sample of Memory Assessment Clinicians in Coventry. Overall, this survey was uptake by 17 clinicians. The pre training survey was done in early October 2019 and the post training survey was done shortly after the training, at the end of October 2019.

A brief training comprising NICE Guidelines and using Confusion Assessment Method (CAM) was delivered. The survey is repeated post training and differences in result of level of confidence is done to measure changes. The survey assessed knowledge, beliefs, practices and confidence level regarding delirium detection.

**Result:**

Pre training:

17 clinicians took part in the survey. 59% was aware that there is a delirium NICE guidelines. 12% felt strongly agree, 41% agree and 47% felt neutral in their confidence of detecting delirium.

Post training:

10 clinicians took part in the survey. 50% felt strongly agree and 50% agree that they are confident in detecting delirium.

Overall, the mean difference is 2 and the p value is 0.92034. we used Mann- Whitney Test to measure the difference in pre and post training which showed not significant at p < 0.05.

Participants felt that the training was useful and relevant to practice.

**Conclusion:**

This study showed our clinicians have a good basic knowledge in detecting delirium. As a result of this study, we have created ‘Delirium checklist’ and Confusion Assessment Method (CAM) to be used during duty work. We also feel that the majority of delirium cases referred to us comes from the community base, thus our next step of the project will be to involve educational work with the community care home.

